# Linear IgA Disease: A Rare Complication of Vancomycin

**DOI:** 10.7759/cureus.4848

**Published:** 2019-06-06

**Authors:** Maryam Saleem, Hassaan Iftikhar

**Affiliations:** 1 Internal Medicine, Waterbury Hospital, Waterbury, USA; 2 Internal Medicine, St. Francis Medical Center, Seton Hall University, Trenton, USA

**Keywords:** vancomycin, linear iga disease

## Abstract

Linear immunoglobulin A (IgA) bullous dermatosis, also known as linear IgA disease, is a rare disorder with an incidence of about 0.5 to 2.3 cases per million individuals per year. In most of the cases, the cause is unknown; however, 50% of the cases are drug-induced. The disease has bimodal age predilection and occurs in children up to the age of 10 years and in adults usually after the age of 60 years. Common drugs that have been known to cause this disease include vancomycin, lithium, amiodarone, captopril, and some of the nonsteroidal anti-inflammatory agents. We report a case of linear IgA disease secondary to vancomycin.

## Introduction

Vancomycin is one of the most common antibiotics used in hospital covering many organisms, including methicillin-resistant *Staphylococcus aureus*. The most common side effects include the red man syndrome, which is characterized by flushing of the face and neck, along with pruritis and sometimes hypotension. Other side effects are mostly gastrointestinal, including nausea, vomiting, and diarrhea, along with nephrotoxicity and ototoxicity [1]. Cutaneous manifestations include Stevens-Johnson syndrome (SJS), toxic epidermal necrolysis (TEN), immunoglobulin E (IgE)-mediated anaphylaxis, and rarely, linear immunoglobulin A (IgA) bullous dermatosis [2-3]. As mentioned earlier, the incidence of linear immunoglobulin A (IgA) bullous dermatosis is not common and there have only been a few reports of IgA bullous dermatosis in the literature [4-8].

## Case presentation

A 73-year-old male with no known drug allergies and a past medical history of diabetes mellitus Type II, hypertension, hyperlipidemia, and bilateral total hip replacements initially presented with confusion and sepsis secondary to right third phalanx osteomyelitis and concurrent *Staphylococcus intermedius* bacteremia. The patient also had a known loose total right hip prosthesis, for which he underwent a right total prosthetic hip removal with the placement of antibiotic-impregnated beads (vancomycin and tobramycin), cement spacer, and wound-vac placement. Intraoperative joint washing demonstrated rare polymorphonuclear neutrophils, gram-positive cocci, and gram-negative rods. The following day, he was noted to have an erythematous rash in his groin which was followed by blisters in the groin, along with uncomfortable burning over his back. Examination revealed at least four to five tense blisters, the largest one was 7 x 7 cm in diameter in the groin with an oozing serous discharge. The initial picture looked like Stevens-Johnson syndrome (SJS), except for the fact it did not involve any mucosa. His medications were reviewed, and all new medications started in the previous 48 hours were discontinued, including rivaroxaban, celecoxib, and ceftriaxone. Vancomycin was continued initially pending intraoperative wound culture results. He was treated with diphenhydramine in the meantime, and general surgery performed punch biopsies of both the groin and back. The next day, the patient developed an erythematous, warm, confluent macular rash that involved the entire back with extension into the buttock and posterior bilateral thighs with continued bullous blisters present in the bilateral groin, some of which had ruptured with serous drainage.

Later on, the patient was found to have extensive warm, erythematous, confluent macular erythema with tense bullae over the distal lower extremities and lateral right abdomen. The initial biopsy results came back as patchy intraepidermal and subepidermal bullae with marked acute and chronic inflammation in the superficial dermis at the epidermal-dermal junction and within the epidermis (neutrophil-predominant) with focal epidermal necrosis/necrolysis, and focal separation and loss of the epidermis, consistent with a blistering skin disorder.

**Figure 1:**
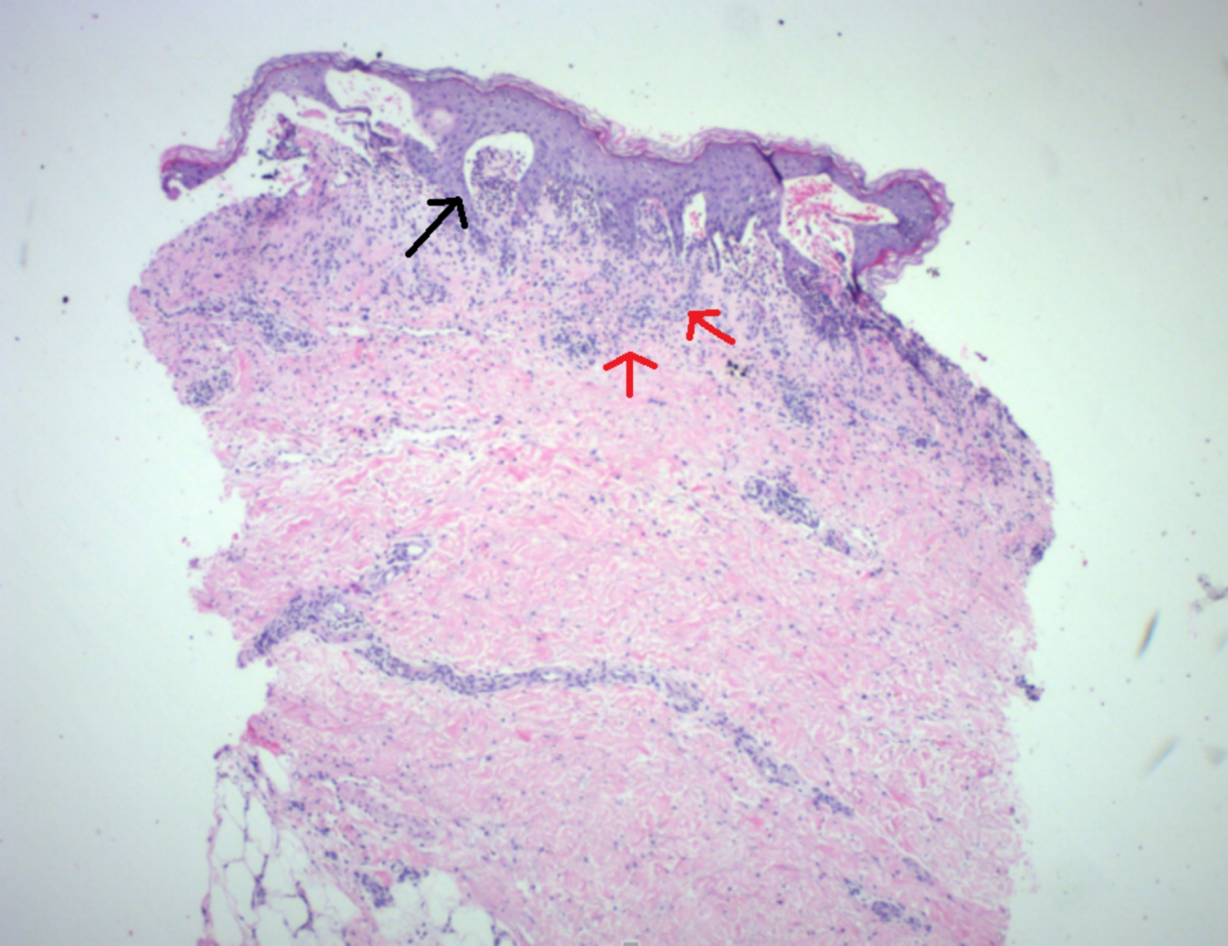
Skin punch biopsy (40x magnification) with superficial inflammation (red arrows) and subepidermal blister formation (black arrow)

**Figure 2:**
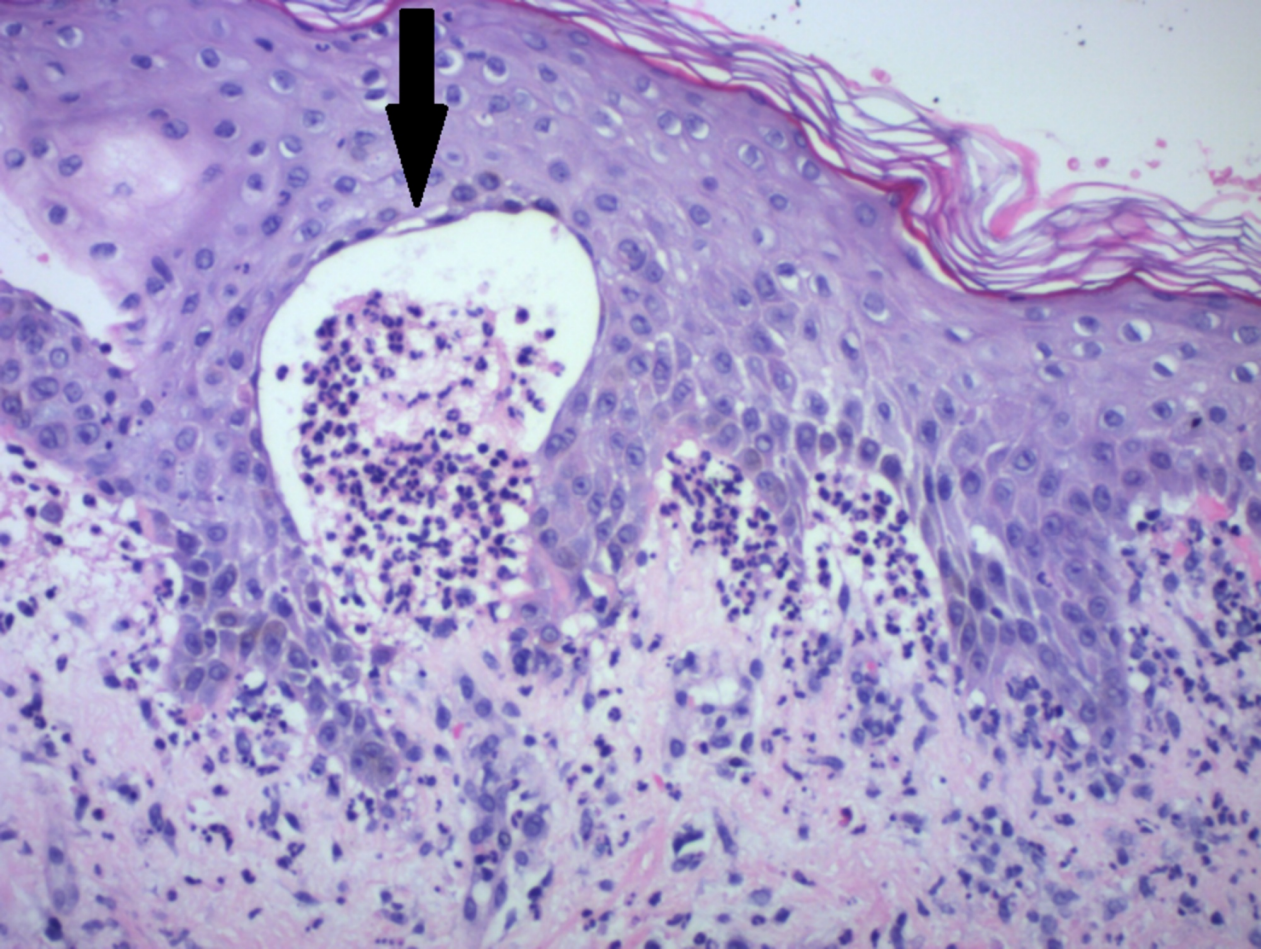
Epidermis (200x magnification) with subepidermal blister formation and neutrophils (arrow)

Figure 1 shows the skin punch biopsy under light microscopy (40x magnification). Figure 2 shows the skin punch biopsy under light microscopy (200x magnification).

Because of worsening rash and new tense bullae formation despite treatment with antihistamines and discontinuing the possible inciting medications, the patient was transferred to a tertiary care center. Initial differentials were morbilliform drug eruption with superimposed edema, bullous drug eruption, linear IgA bullous dermatosis, drug-induced IgA pemphigus, and Stevens-Johnson syndrome. The patient was started on prednisone, 55 mg twice a day (1 mg/kg divided into two doses), along with a topical steroid (clobetasol). Meanwhile, immunofluorescence for the skin biopsy came back positive for IgA, confirming the diagnosis of linear IgA disease. Topical and oral prednisone was continued, along with skin care (Vaseline-soaked gauze over the blisters) with significant improvement. The initial plan was to remove the vancomycin beads from the hip if the patient developed any signs of organ dysfunction which, fortunately, did not happen and the patient recovered well. The patient was discharged home on a prednisone taper over a course of three weeks.

## Discussion

Linear IgA bullous dermatosis (LABD) is an autoimmune disorder which is mainly idiopathic but can also rarely be caused by medications. Vancomycin is the most common drug associated with LABD. Both humoral and cellular immune responses lead to the formation of IgA-1 antibodies that target antigen on the basement membrane. This leads to an inflammatory response with the release of the enzymes by neutrophils that result in tissue injury and leads to skin and mucosal lesions. Skin lesions usually appear one to 14 days after the first dose of vancomycin. This disease initially resembles SJS/TEN in the initial presentation with skin lesions and blisters but the immunofluorescence finding showing a linear pattern is classical for IgA disease. Treatment is usually discontinuing the drug. Medications, including dapsone and high-potency topical steroids (such as clobetasol or betamethasone), are the first-line agents [4-7]. However, colchicine can be used if patients are unable to tolerate dapsone. Severe disease with systemic manifestation may require treatment with systemic corticosteroids and immunosuppressive medications [8]. Treatment is usually continued even after the lesions are resolved; however, this disease may lead to significant morbidity due to mucosal involvement with resultant corneal and mucosal scarring.

## Conclusions

Linear IgA disease is a rare but very severe side effect of vancomycin. The disease mainly involves the skin and mucosa, leading to blister formation. Skin biopsy is usually essential to differentiate it from other blistering disorders, and the diagnosis of linear IgA disease is usually established by immunofluorescence. Treatment usually involves discontinuation of the drug, as well as treatment with steroids or immunosuppressive agents. This case illustrates the importance of early diagnosis of a rare side effect of a commonly prescribed antibiotic to prevent the extension of disease. 
